# Widespread arenavirus occurrence and seroprevalence in small mammals, Nigeria

**DOI:** 10.1186/s13071-018-2991-5

**Published:** 2018-07-13

**Authors:** Ayodeji Olayemi, Akinlabi Oyeyiola, Adeoba Obadare, Joseph Igbokwe, Adetunji Samuel Adesina, Francis Onwe, Kingsley Nnanna Ukwaja, Nnennaya Anthony Ajayi, Toni Rieger, Stephan Günther, Elisabeth Fichet-Calvet

**Affiliations:** 1 Natural History Museum, Obafemi Awolowo University, Ile Ife, Osun State Nigeria; 2 Department of Zoology, Obafemi Awolowo University, Ile Ife, Osun State Nigeria; 3 Department of Biochemistry and Molecular Biology, Obafemi Awolowo University, Ile Ife, Osun State Nigeria; 4 Epidemiology Unit, Ministry of Health, Abakaliki, Ebonyi State Nigeria; 5 Department of Internal Medicine, Federal Teaching Hospital Abakaliki, Abakaliki, Ebonyi State Nigeria; 6 Bernhard Nocht Institute for Tropical Medicine, Hamburg, Germany

**Keywords:** Host, Lassa fever, Rodents, Serology, Zoonotic virus

## Abstract

**Background:**

Lassa fever, killing thousands of people annually, is the most reported viral zoonotic disease in Nigeria. Recently, different rodent species carrying diverse lineages of the Lassa virus (LASV) in addition to a novel Mobala-like genetic sequence were detected within the country. Here, screening 906 small mammal specimens from 11 localities for IgG antibodies and incorporating previous PCR detection data involving the same populations, we further describe arenavirus prevalence across Nigeria in relation to host species and geographical location.

**Methods:**

Small mammals were trapped during the period 2011–2015 according to geographical location (endemic and non-endemic zones for Lassa fever), season (rainy and dry seasons between 2011 and 2012 for certain localities) and habitat (indoors, peridomestic settings and sylvatic vegetation). Identification of animal specimens from genera such as *Mastomys* and *Mus* (*Nannomys*) was assisted by DNA sequencing. Small mammals were tested for LASV IgG antibody using an indirect immunofluorescence assay (IFA).

**Results:**

Small mammals were infected in both the endemic and non-endemic zones for Lassa fever, with a wider range of species IgG-positive (*n* = 8) than those which had been previously detected to be PCR-positive (*n* = 3). IgG-positive species, according to number of infected individuals, were *Mastomys natalensis* (*n* = 40), *Mastomys erythroleucus* (*n* = 15), *Praomys daltoni* (*n* = 6), *Mus baoulei* (*n* = 5), *Rattus rattus* (*n* = 2)*, Crocidura* spp. (*n* = 2), *Mus minutoides* (*n* = 1) and *Praomys misonnei* (*n* = 1)*.* Multimammate mice (*Mastomys natalensis* and *M. erythroleucus*) were the most ubiquitously infected, with animals testing positive by either PCR or IgG in 7 out of the 11 localities sampled. IgG prevalence in *M. natalensis* ranged from 1% in Abagboro, 17–36 % in Eguare Egoro, Ekpoma and Ngel Nyaki, up to 52 % in Mayo Ranewo. Prevalence according to locality, season and age was not, however, statistically significant for *M. natalensis* in Eguare Egoro and Ekpoma, localities that were sampled longitudinally.

**Conclusions:**

Overall, our study demonstrates that arenavirus occurrence is probably more widely distributed geographically and in extent of host taxa than is currently realized. This expanded scope should be taken into consideration in Lassa fever control efforts. Further sampling should also be carried out to isolate and characterize potential arenaviruses present in small mammal populations we found to be seropositive.

## Background

Certain rodent-borne arenaviruses are known to cause illnesses in humans. The deadly Lassa fever in West Africa, with up to 5000 fatalities annually [1], is caused by Lassa virus (LASV) which was recently discovered to be maintained by multiple rodent reservoirs [2]. Etiological agents of hemorrhagic fevers in South America include the Junin virus (borne by the drylands vesper mouse *Calomys masculinus* and other rodents), Machupo virus (borne by the larger vesper mouse *Callomys callosus*) and Guanarito virus (hosted by the common cane mouse *Zygodontomys brevicauda*) [3]. Select cases of meningitis or encephalitis in several countries worldwide are caused by the lymphocytic choriomeningitis virus (LCMV), which is carried by the house mouse *Mus musculus* [4, 5].

Other arenaviruses which are not currently known to be associated with disease in humans continue to be discovered. Across Africa, for instance, Mobala virus was detected in *Praomys* sp. within the Central African Republic [6]; Kodoko virus in *Mus minutoides* within Guinea [7]; and Jirandogo and Natorduori viruses in *Mus baoulei* and *Mus mattheyi*, respectively, within Ghana [8]. These fast-evolving RNA viruses, though apparently non-pathogenic, are also of epidemiological importance as they possess the potential to emerge in new, possibly harmful ways. Indeed, some of them such as the rodent-borne Gbagroube and Jirandogo viruses were demonstrated to be closely related phylogenetically to LASV lineage I [8, 9]. Surveys are therefore needed to evaluate the occurrence of these arenaviruses across host species and at wider geographical scales than previously sampled, such as was recently done in Tanzania, eastern Africa [10].

Prevalence data for zoonotic viruses in relation to parameters such as season, sex and age of host animals also contribute insight into how these viruses are transmitted and maintained between individuals. For example, researchers were able to link human epidemics to seasonal cycles of Puumala hantavirus prevalence in the bank vole *Myodes glareolus* [11]. In instances where there is significant difference in virus prevalence between sexes, male rodents have usually been found to possess higher infection rates possibly due to their propensity to inflict wounds on each other as they fight and display territorial behavior [12]. Age-wise prevalences provide an indication of whether virus transmission is predominantly horizontal or vertical between individual conspecific hosts [13].

Recent studies have made available some information concerning arenavirus occurrence in small mammals within Nigeria: LASV lineage II and a novel Mobala-like virus were detected in the natal multimammate mouse *Mastomys natalensis*, while LASV lineage III was discovered in the Guinea multimammate mouse *Mastomys erythroleucus* and the Kako strain of LASV in the African wood mouse *Hylomyscus pamfi* [2, 14]. Screening antigens and antibodies such as immunoglobin G helps detect acute and previous infections, thus providing further insight into occurrence and prevalence dynamics. Therefore it was our aim in this study to combine PCR detection data from Olayemi et al. [2, 14] with new serological information including the same animals in order to provide further insight into arenavirus occurrence within small mammals in Nigeria.

## Methods

### Small mammal sampling

**Fig. 1:**
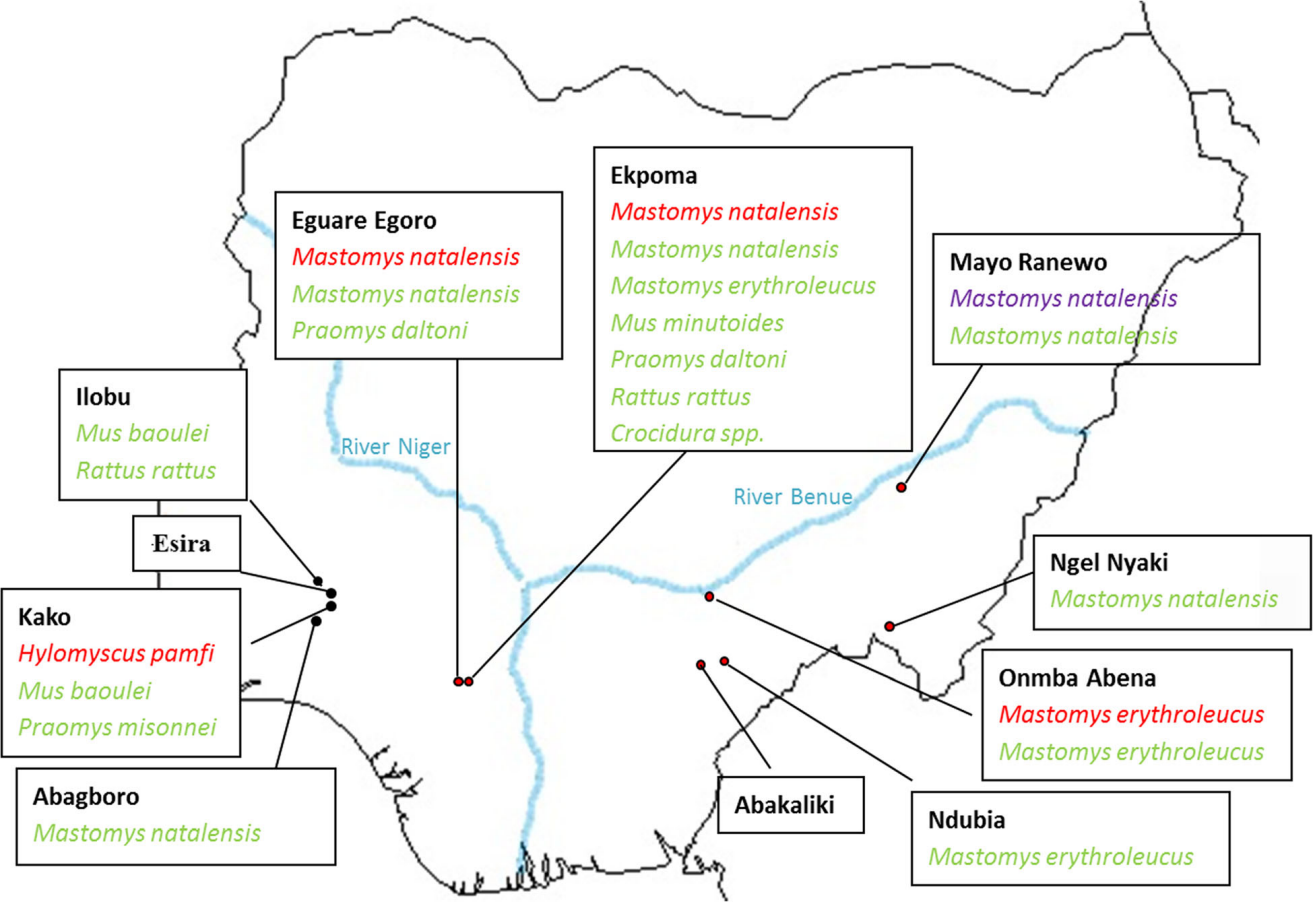
Map of Nigeria showing small mammals that tested arenavirus-positive according to locality. PCR-positive species are in red (indicating Lassa virus) and purple (representing a Mobala-like virus). IgG-positive species are in green. Red dots indicate localities within the endemic Lassa fever zone and black dots mark those outside the endemic zone

A total of 906 small mammal specimens were sampled from 11 localities within Nigeria (Fig. 1, Table 1). Coordinates for each locality are as follows: Abagboro 7°32'38.0"N, 4°30'47.2"E; Kako 7°41'26.3"N, 4°37'09.8"E; Esira 7°42'04.7"N, 4°39'19.4"E; Ilobu 7°52'23.1"N, 4°31'35.4"E; Eguare Egoro 6°46'22.7"N, 6°05'32.5"E; Ekpoma 6°44'29.1"N, 6°06'17.6"E; Mayo Ranewo 8°49'27.2"N, 10°55'15.2"E; Ngel Nyaki 7°05'30.8"N, 11°05'7.9"E; Onmba Abena 7°38'27.5"N, 8°24'23.6"E; Ndubia 6°21'45.9"N, 8°19'22.7"E; and Abakaliki 6°17'38.9"N, 8°5'54.3"E. Small mammals from 9 of these localities were trapped between 2011 and 2013. Through this period, Abagboro, Eguare Egoro and Onmba Abena were investigated during the dry season (January-March) and rainy season (September-October) in both 2011 and 2012. Ekpoma was investigated in the dry season and rainy season in 2012 only. In addition to these 9 localities, trapping was conducted in 2 sites within eastern Nigeria (Abakaliki and Ndubia) during November 2015. Based on the published research literature [15–20] 7 of the 11 localities sampled are regarded as sited within the endemic area for Lassa fever within Nigeria, where epidemics are frequent (Fig. 1, Table 1).

**Table 1:** Small mammals trapped during the study with number of arenavirus-positive individuals (PCR-positive/IgG-positive). For *Mastomys erythroleucus* and *M. natalensis* additional numbers in parentheses indicate (PCR percentage prevalence/IgG percentage prevalence)

	Non endemic zone for Lassa fever				Endemic zone for Lassa fever							Total
	Abagboro	Ilobu	Kako	Esira	Eguare Egoro	Ekpoma	Abakaliki	Ndubia	Onmba Abena	Mayo Ranewo	Ngel Nyaki	
Rodentia												
*Aethomys* cf. *stannarius*									2			2
*Arvicanthis niloticus*						1				3		4
*Gerbilliscus kempi*									12			12
*Hylomyscus pamfi*	3		2 (1/-)									5
*Lemniscomys striatus*									3			3
*Lophuromys sikapusi*	1			1	2	2						6
*Mastomys erythroleucus*						2 (-/1) (- /50%)	31	20 (-/3) (- /15%)	63 (3/11) (5%/18%)			116
*Mastomys natalensis*	99 (-/1) (- /1%)		7	13	29 (6/5) (21%/17%)	36 (9/13) (25%/36%)			34	31 (1^a^/16) (3%/52%)	24 (-/5) (- /21%)	273
*Mus baoulei*	6	5 (-/4)	4 (-/1)									15
*Mus minutoides*	12			1	1	3 (-/1)			2			19
*Mus musculoides*								5	4			9
*Mus setulosus*	6		9		1						1	17
*Praomys daltoni*	12			2	126 (-/4)	47 (-/2)						187
*Praomys jacksoni*											16	16
*Praomys misonnei*			1 (-/1)									1
*Rattus rattus*		3 (-/1)			1	3 (-/1)	3	4	6			20
*Uranomys ruddi*					1							1
Insectivora												
*Crocidura spp.*	14		4	3	19	69 (-/2)	24	29	32	4	1	199
*Erinaceus albiventris*										1		1
Total	153	8	27	20	180	163	58	58	158	39	42	906

^a^PCR-positive results in all cases represent LASV infection except in Mayo Ranewo, where *M. natalensis* was PCR-positive for a Mobala-like arenavirus

Adapted from [2, 14, 20]

With regard to sampling habitats, trapping was varied slightly according to whether a locality was relatively rural or urban. Almost all the localities sampled in this study (9 out of 11) are villages in a rural setting (or villages on the outskirts of a larger town). In these areas, trapping indoors (within kitchens, bedrooms and food storage areas) was carried out through a transect running across the village, with 2 traps dispatched per room. Trapping outdoors was carried out in habitats far removed from immediate human habitation (sylvatic vegetation such as forest, savanna or fallow land) in trap-lines containing 20 traps spaced over 100 m with 5 m between individual traps. The two remaining localities (Ekpoma and Abakaliki) are considered relatively urban in terms of size and presence of modern civic infrastructure. Here, instead of a transect, trapping was carried out indoors in various house addresses dotted across the city. In this case, sampling outdoors was in a peridomestic setting: in gardens (sometimes littered with rubbish heaps) not far from human habitations. Therefore, overall, 3 habitat types were sampled in this study: indoors, peridomestic vegetation and sylvatic vegetation. The Osun State Ministry of Environment, the Taraba State Ministry of Health, and the Gwer West Local Government Area office in Benue State granted permission to trap small mammals in the localities under their authority.

### Small mammal identification

Morphological identification of small mammals was done using external criteria such as fur color, nipple formula, and standard measurements: body weight, head-and-body length, tail length, hindfoot length and ear length [21]. Molecular identification was facilitated by DNA cytochrome *b* sequencing, especially for the *Mastomys* spp. and other sibling species such as those within *Mus* (*Nannomys*) spp. We did not designate specimens from *Crocidura* to species level because, even with the benefit of cytochrome *b* sequencing, the taxonomy of this genus is not well established. For example, the species currently known as *C. olivieri* (for which a large number of specimens were morphologically identified in our collection) has very recently been described as polyphyletic [22]. Detailed information concerning trapping methods and the species composition appears in [20].

### Serology and PCR screening methods

Small mammals were tested for Lassa virus (LASV) IgG antibody using an indirect immunofluorescence assay (IFA). As LASV and other closely related arenaviruses are known to cross-react serologically, IgG-positive samples were interpreted as being indicative of a past infection by LASV or possibly some other arenavirus. Whole blood was diluted at 1:20 in 1% triton-phosphate buffer saline (PBS) solution. Serum from the liver rinsed with 1% triton-PBS was used when blood was not available. Seven microliter drops of this solution were then transferred unto slides spotted with the Bantou strain of LASV grown beforehand in Vero cells (see details of the method in [23]). Samples showing fluorescence were regarded IgG-positive. Positive samples were confirmed by a second independent viewing. Samples with ambiguous results were further examined on a slide that had the upper row of spots infected with the LASV antigen and the lower row non-infected. Side-by-side comparison of infected and non-infected spots bearing the same sample enabled an eventual decision concerning IgG status.

Total RNA was extracted from 20 μl of whole blood using a QIAamp Viral RNA Mini kit (Qiagen, Valencia, CA, USA). Using a Qiagen RT-PCR kit, extracted RNA was tested with LASV-specific primers on the GPC gene [24] and with pan-arena primers on the L gene [25].

### Statistical analyses

The weight in milligrams of the eye lens (ELW), dried in an incubator for 2 h at 100 °C, was used as a surrogate for small mammal age [26–28]. The statistical analysis was restricted to Eguare-Egoro and Ekpoma, two localities that were sampled longitudinally and where *M. natalensis* had both PCR- and IgG-positive individuals. The effects of locality (2 levels: see above), season (2 levels: dry, rainy) and age (log-transformed ELW as a continuous variable) on arenavirus infection were evaluated by logistic regression using a generalized linear model with logit-link function in R software [29]. Regressions were performed for each infection, IgG or antigen, including locality and season as fixed effect. We kept the variable “locality” as a fixed effect because one was rural and the other one was periurban. These localities were designated because of the presence of human Lassa cases, and were not chosen randomly. As our data set is small (*n* = 65), we introduced only one 2-way interaction “locality × season”. This interaction was chosen because of possible spatial and temporal risk of LASV transmission from rodent to humans. In order to remain in the conditions of validity of the test which stipulates to have at least 5 events by explanatory variable, we kept only 3 independent variables: the locality, the season and the age [30].

## Results

PCR- and IgG-positive small mammals were detected in both endemic and non endemic zones for Lassa fever (Fig. 1). Overall, PCR and IgG infection were most ubiquitous in *M. natalensis* (16 and 40 individuals, respectively; Table 1). This species tested positive in 5 out of 8 localities where the rodent was trapped. Out of the 5 localities, Eguare Egoro and Ekpoma contained individuals PCR-positive for LASV while Mayo Ranewo contained a Mobala-positive individual. Next in IgG occurrence, *M. erythroleucus* was positive in 3 out of 4 localities (15 individuals). In one of these localities (Onmba Abena) 3 *M. erythroleucus* individuals were also LASV-positive.

Apart from *M. natalensis* and *M. erythroleucus*, 6 other small mammal taxa were also IgG positive: *Praomys daltoni* (*n* = 6 individuals), *Mus baoulei* (*n* = 5), *Rattus rattus* (*n* = 2)*, Crocidura* spp. (*n* = 2), *Mus minutoides* (*n* = 1) and *Praomys misonnei* (*n* = 1). *Praomys daltoni*, *P. misonnei* and *Crocidura* spp. were only seropositive in the localities where *M. natalensis* and *H. pamfi* were LASV-positive. The remaining species were seropositive in both LASV-positive and LASV-negative localities (Fig. 1, Table 1).

**Fig. 2:**
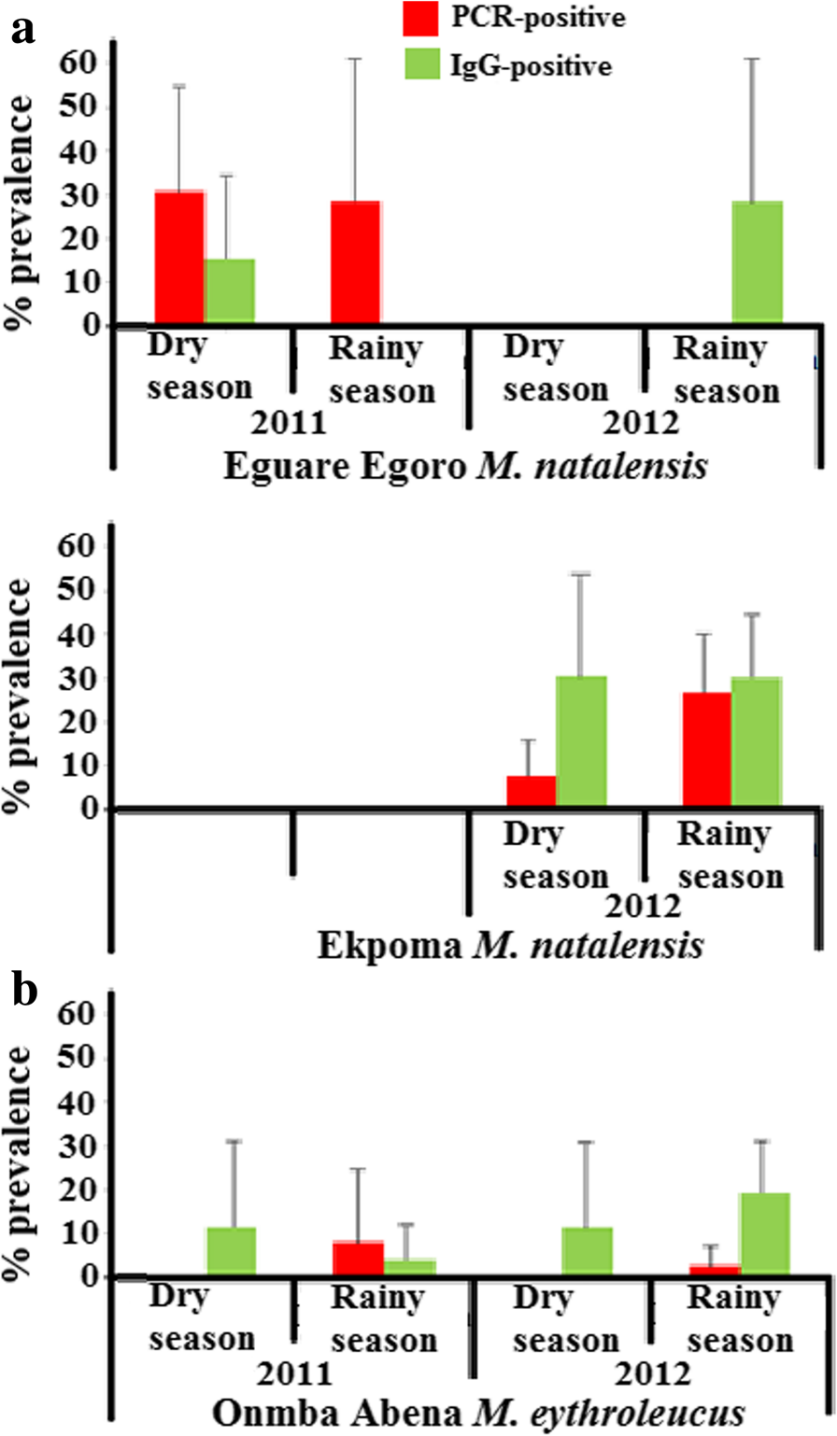
PCR and IgG prevalence in *Mastomys natalensis* (**a**) and *M. erythroleucus* (**b**) according to season (with 95% confidence intervals)

**Fig. 3:**
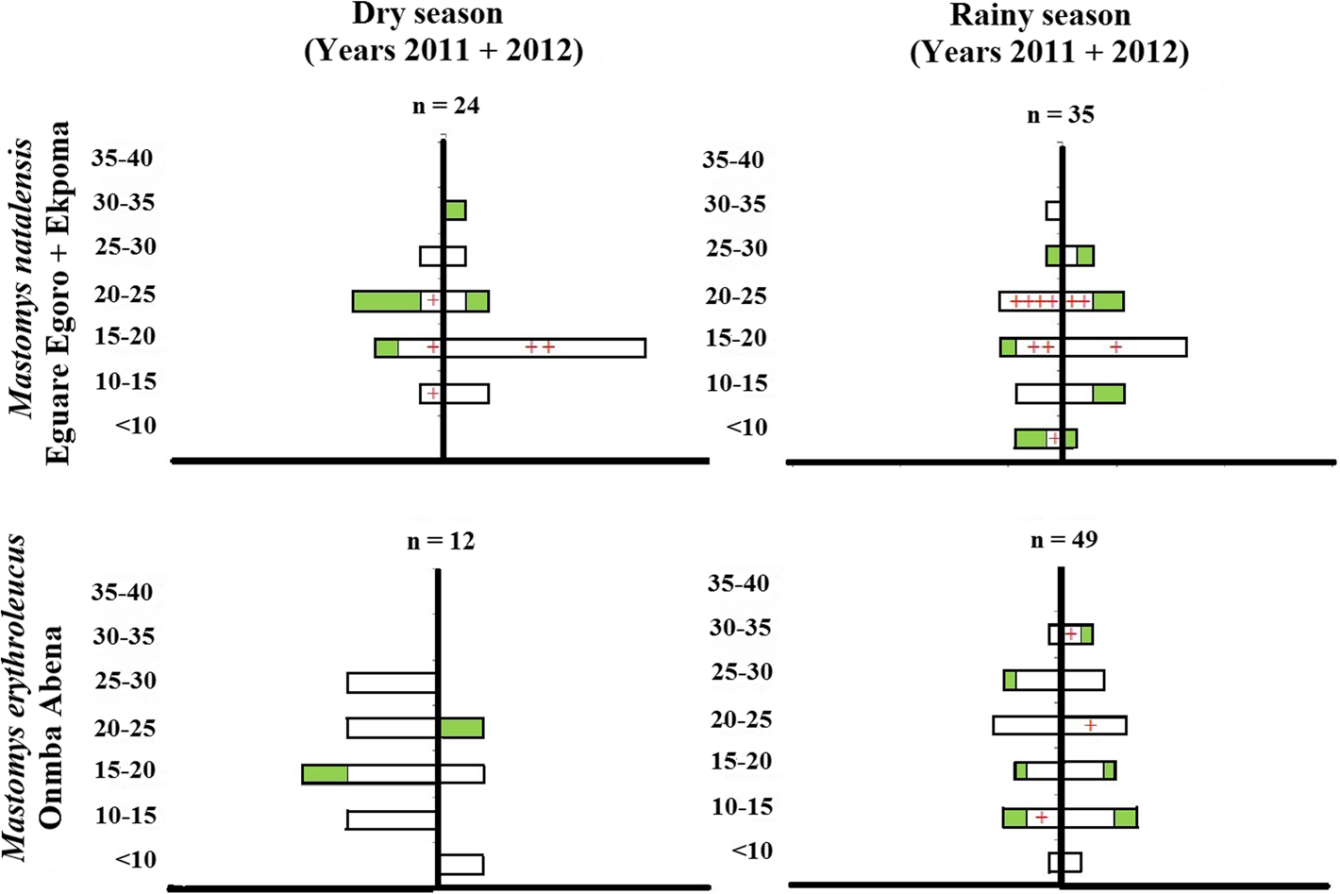
Arenavirus infection status by sex and age category in *Mastomys* populations that were sampled longitudinally. White bars represent IgG-negative individuals, green bars IgG-positive individuals, and red crosses indicate the number of LASV PCR-positive individuals in a particular age category. Age categories are represented by eye lens weight in milligrams on the dependent axes. Bars to the left of each independent axis represent female individuals, those on the right represent male individuals. Each pyramid was scaled so that the surface area of the bars total 100 %

Of all the individual small mammals captured and tested in this study, none were PCR- and IgG-positive at the same time. Because arenavirus infection was most extensive in *Mastomys natalensis* populations, we attempted to examine variation in prevalence according to locality, season, and age in this species. However, our analyses did not show prevalence for any of these variables to be statistically significant (Table 2). Although not statistically significant, PCR and IgG distribution for key *Mastomys* populations are depicted by season (Fig. 2), sex and age (Fig. 3).

**Table 2:** Probability values (*P*) from statistical comparison by logistic regression of arenavirus prevalence in *M. natalensis* in Eguare Egoro and Ekpoma (EKP)

Variable		PCR prevalence (*n* = 65)		IgG prevalence (*n* = 65)	
	*df*	Estimate	*P*	Estimate	*P*
(Intercept)		-3.1733	0.318	-1.8615	0.527
Age (eye lens weight in mg)	1	1.6387	0.488	0.3608	0.869
Locality (EKP)	1	-1.2167	0.313	0.8444	0.351
Season (rainy)	1	-0.7367	0.445	-0.3942	0.694
Locality (EKP) × season (rainy)	1	2.3431	0.116	0.3912	0.755

## Discussion

### Arenavirus occurrence

Arenavirus occurrence (as detected by PCR and IgG markers) was widespread across the study area in various small mammal species but was most ubiquitous in *Mastomys natalensis* and *M. erythroleucus*. Out of seven non-*Mastomys* small mammal species that were IgG positive, four (*Rattus rattus*, *Praomys daltoni*, *Mus minutoides* and *Crocidura* spp.) occurred where infected *M. natalensis* were also present. In Guinea, non-*M. natalensis* species were also detected that were IgG-positive, including *P. daltoni* and *Mus minutoides* (as in this study) but also *P. rostratus* and *Lemniscomys striatus* [31]. Up to now, active LASV infections (by virus isolation or PCR) have never been detected in these species [2, 32, 33]. Their IgG antibodies probably indicate temporary, spillover infections caused by their proximity to infected *Mastomys* in areas highly endemic for Lassa fever like Eguare Egoro and Ekpoma.

Four non-*Mastomys* were also arenavirus-postive in Kako and Ilobu, outside the endemic zone for Lassa fever (Fig. 1). These are *Hylomyscus pamfi* (PCR-positive), *Mus baoulei*, *Praomys missonei* and *Rattus rattus* (all IgG-positive). *H. pamfi* in Kako was recently discovered to be the natural reservoir of a LASV strain close to lineage I. The IgG signatures detected in *Mus baoulei* and *P. missonei* in Kako could represent spill-over infections from *H. pamfi*. However, in Ilobu there is a high IgG prevalence in *Mus baoulei* (4/5). Interestingly, in nearby Ghana, *Mus baoulei* was also recently detected to be host of the Jirandogo arenavirus [8], which like the lineage found to infect *H. pamfi*, is close to LASV lineage I. Further sampling in Kako and Ilobu will provide increased insight into evolution of LASV in non-*Mastomys* in the non endemic area of Lassa fever in south-western Nigeria.

### Arenavirus infection in *Mastomys*

The geographical disparity in seroprevalence for *M. natalensis* was as high as 52% in Mayo Ranewo, 17–36% in Eguare Egoro, Ekpoma, Ngel Nyaki, and as low as 1% in Abagboro. IgG positive *M. natalensis* individuals in populations where there were also LASV-PCR-positive mice (Eguare Egoro and Ekpoma) probably indicate previous LASV infections. Similarly, the IgG-positive *M. natalensis* in Mayo Ranewo probably reflect past infection by the Mobala-like arenavirus detected by PCR in this same rodent species.

IgG signatures in *M. natalensis* populations that were not PCR-positive (e.g. Ngel Nyaki) might well represent LASV or some other arenavirus, as antibodies of closely related arenaviruses are known to cross-react with the LASV antigen used in this study [31]. Indeed, in various parts of Africa *M. natalensis* is a well known carrier of arenaviruses other than LASV such as Mopeia [34, 35], Luna [36, 37] and Gairo [38] viruses.

For *M. erythroleucus*, seropositive individuals detected beyond Onmba Abena (where it was only recently detected to be a LASV host [2]) present the possibility this rodent may be more involved in Lassa fever epidemiology in areas around Ndubia [39] and Ekpoma [20] than is currently realized.

PCR and IgG prevalence were not significantly different between seasons for *Mastomys natalensis* in populations sampled longitudinally in this study. On the other hand, a significantly higher LASV prevalence was found in *M. natalensis* during the rainy season within Guinea [40]. Failure to obtain statistically significant results for season in this study may have been due to our relatively small sample size (*n* = 65 for *M. natalensis* in Eguare and Ekpoma compared to *n* = 553 for *M. natalensis* in Guinea [40]).

There was also no statistical difference in arenavirus prevalence (antigen or antibody) in age within *M. natalensis*. However, the presence of both PCR- and IgG-positive individuals in the youngest age class of *M. natalensis* in Ekpoma supports previous findings that LASV [41] and other arenaviruses such as Mopeia [42] are transmitted through both vertical and horizontal modes.

Combined detection of arenavirus prevalence in small mammals in this study by PCR (representing active infection) and IgG (indicating previous infection) contributes to data already presented in other studies concerning seroconversion [31, 43, 44]. These studies demonstrate that arenavirus infections in small mammals are normally acute, with the virus clearing quickly to be replaced by antibodies. Therefore, individuals tested usually are either only PCR-positive or IgG-positive, with very few individuals carrying these two markers of infection at the same time. The acute infection model was supported in this study as no individual was jointly PCR- and IgG-positive in *Mastomys* (or any other small mammal tested). This pattern is best illustrated by the Mobala-like arenavirus infection of *M. natalensis*, which had the highest IgG prevalence (52%) among all positive *Mastomys* populations but, conversely, the lowest PCR prevalence (3%). However, although virus was absent from blood in IgG-positive individuals, our data does not exclude the possibility that seropositive animals were actively excreting LASV in urine or saliva or contained latent virus in the organs.

## Conclusions

Our findings reveal arenavirus infection has a much expanded geographical distribution and small mammal host range than has been discovered within Nigeria in the past. Populations of *Mastomys natalensis*, *M. erythroleucus* and *Hylomyscus pamfi*, being natural hosts of LASV and a Mobala-like virus, included PCR- and IgG-positive individuals, but several other small mammal species were only IgG-positive. Among the animals exclusively IgG-positive could be natural hosts of other yet-to-be-described arenaviruses or incidental hosts caused by spill-over infections. However, even spill-over infections must be accorded epidemiological importance as challenge by continually evolving viruses may lead to adaptation and eventual emergence in new small mammal species [45]. Further attempts to isolate and characterize arenaviruses should be made in localities where IgG but not PCR-positive animals were detected. In addition, longer investigations (beyond two years as was the scope in this study) and increased sampling (between and within seasons) should be carried out to understand more fully the dynamics of Lassa and other arenaviruses borne by small mammals in order to counter their zoonotic effect in Nigeria.

## Abbreviations

DNA: deoxyribonucleic acid
ELW: eye lens weight
LASV: Lassa virus
